# Fitspiration and thinspiration: a comparison across three social networking sites

**DOI:** 10.1186/s40337-018-0227-x

**Published:** 2018-11-26

**Authors:** Angela S. Alberga, Samantha J. Withnell, Kristin M. von Ranson

**Affiliations:** 1 Department of Health, Kinesiology & Applied Physiology, Concordia University, 7141 Sherbrooke Street West; Office: SP-165.06, Montreal, QC H4B1R6 Canada; 2 Department of Psychology, University of Calgary, 2500 University Drive N.W, Calgary, AB T2N 1N4 Canada; 3 Department of Psychology, University of Calgary, 2500 University Drive N.W, Calgary, AB T2N 1N4 Canada

**Keywords:** Social media, Thinspiration, Fitspiration, Fitness, Body image, Eating disorders, Appearance

## Abstract

**Background:**

Fitspiration, or images and text promoting health and fitness, and thinspiration, or images and text promoting thinness, have both received criticism for their negative effects on body image and dieting behaviors. In this study, we critically examined and compared the content of fitspiration and thinspiration on three social networking sites (SNS).

**Methods:**

Fitspiration and thinspiration posts (*N* = 360) from three photo-sharing SNS (Instagram, Tumblr, and Twitter) were collected quasi-randomly on four days over two weeks. Image and associated text content were coded for variables related to weight and shape, muscularity, thin ideal, and eating. Chi-square and Fisher’s exact tests compared content of fitspiration and thinspiration posts overall and among the three SNS.

**Results:**

Thinspiration images portrayed body parts more frequently than fitspiration (69.8% vs. 30.2%). Similarly, posts highlighting bony body features and references to mental illness appeared only in thinspiration. No differences were found between fitspiration and thinspiration posts with regard to sexual suggestiveness, appearance comparison, and messages encouraging restrictive eating. Fitspiration and thinspiration posts included similar images across the three SNS—focusing on appearance, sexually suggestive images, and restrictive eating—with three exceptions. Fitspiration posts exhibiting body positivity were found only on Tumblr. In thinspiration posts, references to mental illness were more frequent on Tumblr and Instagram than on Twitter, and bone emphasis was coded more frequently on Twitter than on Instagram.

**Conclusions:**

Although fitspiration posts were less extreme than thinspiration posts on the whole, notable similarities in their content support that fitspiration endorses problematic attitudes towards fitness, body image, and restrictive eating in pursuit of a fit-and-thin body ideal.

## Plain English summary

We analyzed images from three social media sites (Instagram, Tumblr and Twitter) to describe and compare the content of fitspiration (images and text promoting health and fitness) and thinspiration (images and text promoting thinness) posts. Overall, the fitspiration and thinspiration content of posts was similar across the three social media sites studied, with three exceptions. Thinspiration posts showed more images showcasing bony body parts and mental illness. Both thinspiration and fitspiration images reinforced body image issues and restrictive eating. Thinspiration posts included mention of mental illness more often on Tumblr and Instagram than on Twitter. We conclude that fitspiration showed problematic image content similar to thinspiration in emphasizing a fit-and-thin body ideal.

## Comparing fitspiration and thinspiration content on three social networking sites

Mass media has been identified as an influential cause of body dissatisfaction in women [1, 2]. In recent years, social networking sites (SNS) such as Facebook, Instagram, Twitter, Tumblr, and MySpace have been the subject of much investigation to determine their positive and negative impacts on body image [3, 4]. A systematic review of 20 studies (including 16 cross-sectional and four experimental designs) showed that overall time spent on SNS is associated with body image disturbances and disordered eating [5]. *Thinspiration*, or inspirational messages promoting thinness, has received criticism for its detrimental effects on body image [6]. Existing research has analyzed thinspiration content on SNS platforms such as Facebook, MySpace, Twitter, Pinterest, and Instagram [7–9]. These content analyses have found that thinspiration posts feature images of extremely thin or underweight women, often in sexually suggestive poses focused on the pelvis and abdomen, and the bony features of those parts. Thinspiration content may also contain references to other mental health problems, such as depression, suicide and self-harm [9]. Additionally, researchers [7] have found differences in the severity of thinspiration content between SNS and the hashtags used to identify content.

SNS have also become places to post messages and images “intended to inspire people to live healthy and fit lifestyles through motivating exercise- and diet- related images and text” [10]. Such content is referred to as *fitspiration*. Researchers have begun examining the content of fitspiration on websites as well as SNS [5, 10–17], and have found that fitspiration posts emphasize appearance and attractiveness, rather than health, as motivation for engaging in fitness behaviors. Female subjects in fitspiration images are frequently thin and sexually objectified [10, 13, 17]. Additionally, Boepple et al. [10] found that 45% of fitspiration images included figures posed to appear thinner or smaller than reality (e.g., positioning the camera from above or tilting the hips to minimize body size). These findings suggest a problematic emphasis on thinness and physical attraction as the motivation and reward for exercise and suggest that the female body ideal has shifted to emphasize both extreme thinness and fitness. However, it is still unclear if fitspiration content warrants as much concern as thinspiration.

There is evidence that, like thinspiration, fitspiration content may be detrimental to the mental health of its users. For example, Hefner et al. [18] found that use of fitness-related mobile phone applications and SNS use in general were significantly associated with disordered eating and compulsive exercise behavior. In a comparison of women who posted fitspiration versus travel images on Instagram, Holland and Tiggemann [5] showed greater disordered eating, drive for thinness, and compulsive exercise among women who posted fitspiration images. Although fitspiration content has been shown to increase users’ intentions to improve their fitness, it also decreased body satisfaction and appearance self-esteem, and increased drive for thinness [19, 20]. These findings suggest that fitspiration content has mixed effects on users, simultaneously encouraging health in its promotion of physical exercise as well as harmful attitudes towards eating and the body. Other studies have shown that women exposed to thin-athletic models experience more body dissatisfaction than when exposed to normal-weight-athletic models, neutral objects, and even traditional thin-ideal images [21, 22]. These findings suggest that the addition of fitness to the traditional thin-ideal may have the effect of making the ideal body even more unattainable for women. Further, Homan & Tylka [23] found that positive effects of physical exercise on increasing body appreciation and body satisfaction were weakened when exercise was primarily motivated by a desire to change the appearance of one’s body shape or weight. Supporting a conceptual overlap between fitspiration and thinspiration content, exposure to fitspiration content on Pinterest has also been found to predict willingness to engage in extreme weight-loss tactics, such as crash dieting [24]. Finally, a study of user engagement with online health and fitness content found that participants who also reported misuse of diet pills or other eating disorder symptoms were more likely to ‘like’ fitness-related posts on social media [25].

Given emerging evidence of overlap between thinspiration and fitspiration, directly comparing both types of SNS content is worthwhile to ascertain their similarities and differences. Boepple and Thompson [11] compared images from 50 thinspiration and 50 fitspiration websites, finding that whereas thinspiration featured a greater emphasis on weight loss and thinness, the two types of content did not differ in their emphasis on objectification, dieting, and guilt about body weight or shape. However, the images assessed were from dedicated websites, which may not be as broadly used for photo-sharing as SNS. In contrast, Talbot and colleagues [26] analyzed 734 thinspiration, fitspiration, and “bonespiration” (content glorifying skeletal bodies suggestive of anorexia nervosa) images on three SNS: Twitter, Instagram, and WeHeartIt. They found that thinspiration and bonespiration contained more thin, objectified bodies than fitspiration, which featured more muscular bodies. However, thinspiration and fitspiration did not differ in the frequency of bone protrusions featured in images, suggesting that it may be a subtype of fitspiration posts that is similar to thinspiration. Talbot et al. [26] coded images for only body type and objectification (defined as the proportion of body features visible in the image, with images focused on smaller proportions of features considered more objectifying). They did not examine the text associated with SNS image captions, such as for weight/shape guilt or dieting messages.

Although studies have examined fitspiration and thinspiration content on a variety of SNS, only one [7] has made direct comparisons between platforms. No study to date has compared fitspiration posts between SNS; instead, studies have analyzed fitspiration content drawn from multiple SNS together as a single group. Given that Ghaznavi and Taylor [7] found differences in severity of thinspiration posts between Pinterest and Twitter, it is worth examining whether any SNS differences exist in thinspiration or fitpsiration posts. There is also evidence of differences among user communities of SNS: wealthier teens are more likely to use platforms such as Twitter and Instagram than lower-income teens, and SNS emphasizing photo-sharing, such as Tumblr and Instagram, are more popular with teenage girls than boys [27]. Similarly, to date only Ghaznavi and Taylor [7] have compared content between thinspiration hashtags (tags added to posts which allow users to find or follow specific types of content), and no study to date has compared fitspiration across SNS or hashtags. Comparing content between platforms and hashtags could provide a more complete picture of thinspiration and fitspiration communities on SNS, and may identify subtypes that are most harmful. Finally, most studies have examined only a few image or text-based variables among fitspiration or thinspiration content, leaving gaps in understanding the context in which users are posting images and writing accompanying text captions.

The purpose of the present study was to conduct a content analysis of fitspiration and thinspiration posts on three popular SNS—Instagram, Tumblr, and Twitter—and to examine similarities and differences between fitspiration and thinspiration content.

Based on previous findings [10, 11, 16, 17, 19, 26], we hypothesized that fitspiration would include fewer posts featuring extreme thinness (i.e. emphasizing bony features such as hip and collarbone protrusions) or references to disordered eating symptoms than thinspiration, but would emphasize weight loss, dieting and appearance-based motivations in a manner similar to thinspiration.

A secondary objective of this study was to explore possible differences in fitspiration and thinspiration posts among three SNS, as well as among main hashtags used to identify content in posts. Users may select different SNS according to the features offered (such as emphasis on photo-sharing), or what is popular among their peer group. There are also important demographic differences among users of Instagram, Tumblr and Twitter. For example, almost three-quarters of teens and young adults aged 13 to 24 have reported using Instagram, whereas Twitter and Tumblr are less widely used, with 40% and 16% of teens and young adults reporting having used the platforms respectively [28]. We had no specific hypotheses about the direction or size of any content differences on Twitter, Tumblr and Instagram.

## Methods

### Selection of websites

We chose three photo-sharing SNS (Instagram, Tumblr and Twitter) for their emphasis on photographic content, and because they allowed public access to images through hashtag searches. Based on a previous study that compared fitspiration and thinspiration [11], we investigated the hashtags *#fitspiration* and *#thinspiration* and their most popular variations, *#fitspo* and *#thinspo*. Due to search constraints on Instagram that prevent users from viewing #thinspiration and #thinspo posts, we identified alternative hashtags by typing “thin” and “fit” into the Instagram search bar and selecting the two variations with the most posts *(#thinstagram* and *#thinspoooo*; see Table 1 for summary).

**Table 1:** Hashtags searched for each social networking site

Social media site			
	Instagram	Twitter	Tumblr
#fitspiration	X	X	X
#fitspo	X	X	X
#thinspiration		X	X
#thinspo		X	X
#thinstagram	X		
#thinspoooo	X		

Note: Due to search constraints on Instagram, #thinspiration and #thinspo were not available for searching. Instead, #thinstagram and #thinspoooo were identified as the most common, and therefore were used for our searches on Instagram

Hashtag searches were performed over a two-week period on Instagram, Tumblr, and Twitter in March 2016. Images were collected quasi-randomly, i.e. on Tuesdays and Fridays at similar times of day (11 am to 2 pm). We chose Tuesday and Friday as there are popular hashtags specific to those days (e.g., #TransformationTuesday and #FitnessFriday), which we speculated might increase the volume of new posts. For each hashtag searched, we took a screenshot with a timestamp for each of the most recent fifteen posts with images on each day (30 images total for Tuesday and Friday). Instagram would not display the most recent images for #thinspoooo, and so only the most popular posts were collected for that hashtag.

### Content analysis and ratings of SNS posts

We conducted a content analysis, defined as “a research method for the subjective interpretation of the content of text data through the systematic classification process of coding and identifying themes or patterns” [29], which has been commonly used in other studies that have analyzed fitspiration and thinspiration on social media [8–13] . For this paper, we systematically evaluated qualitative data (images, text and hashtags) of posts from three social networking sites and coded this content into quantitative data to identify themes between fitspiration and thinspiration. We created a coding scheme based on previous research in the field as well as new codes to compare fitspiration and thinspiration (i.e. do posts suggest weight-related issues, mental illness, muscular ideals) that have been previously identified [7, 11]. Posts were rated using a coding scheme that assessed weight/shape-related messages, thin-ideal messages, muscularity, eating-related messages, as well as a variety of descriptive variables (see Table 2). Variables within the weight/shape, thin ideal, and food categories were adapted from previous research [7, 11]. Variables assessing the emphasis on muscularity were added to capture “muscular ideal” in fitspiration posts, similar to “thin ideal” in thinspiration posts identified from previous studies [11]. For example, we added a “muscular pose” category to capture images in which pose and camera angle were used to emphasize one’s muscularity, as opposed to thinness (e.g. posing the arm and flexing the bicep to emphasize muscle). We also added a variable assessing the explicit mention of mental illness in posts, such as eating disorders (e.g. pro-anorexia and pro-bulimia hashtags like #ana, #mia), depression, or suicidality. This variable was added to more systematically capture a content group previously identified by Ging & Garvey [9] in which users tag posts describing suicidal thoughts or desires to self-harm with thinspiration-related hashtags.

**Table 2:** Descriptions of Coded Variables and Inter-Rater Reliability

Variable	Description	Cohen’s Kappa (*κ)*
Descriptors		
Quote	Presence of inspirational quote or message on the image	.84
Language	Text on or below image, or in hashtags contains all or partly English	.94
Image Description	Image contains women and/or men, an object or a graphic	.95
Weight/Shape		
Whole Body	Entire body is visible in the image	.86
Head	Head is visible and unobscured in image	.93
Eyes	Eyes are visible and unobscured in image	.97
Torso	Torso is visible in image	.98
Pelvis	Pelvis is visible in image	.95
Legs	Legs are visible in image	.98
Arms	Arms are visible in image	.95
Appearance Comparison	Comparing body pre- and post-weight loss	.96
Suggestive Pose	Pose emphasizing sex characteristics	.84
Revealing Clothing	Explicitness of attire worn in image	.72
Body Guilt	Expresses guilt for having gained weight, not meeting weight or fitness goals or ideal body type	.90
Weight Loss	Emphasizes losing fat or weight	.88
Body Positive	At least one element of image or text is body positive	.72
Muscularity		
Muscular Pose	Flexing, posing to appear more muscular	.89
Physical Activity	Person engaged in exercise or physical activity	.92
Muscle Emphasis	Prominent focus on muscular features	.75
Thin Ideal		
Thin Pose	Posing or positioning camera to appear thinner or smaller	.67
Thin Praise	Complements for thin bodies, thinness as a marker of success	.76
Bone Emphasis	Prominent focus on bony features such as hip and collarbone protrusions with the absence of defined muscle	.86
Food		
Reducing Food	Messages encouraging reduction of eating	.95
Food Guilt	Guilt for eating certain foods	.96
Mental Illness	Mentions eating disorder, self-harm, anxiety, suicide or depression	.93

We collected and coded a total of 360 posts containing images. Not all hashtags were available on all SNS, resulting in 120 posts from each SNS. Thirty posts were collected from four hashtags on each SNS (#thinspiration, #thinspo, #fitspiration, and #fitspo on Tumblr and Twitter; #fitspiration, #fitspo, and the alternative hashtags #thinstagram and #thinspoooo on Instagram). Posts were excluded if they were not in English or French (analysts were fluent in only these two languages), if they were pornographic (i.e. if the person was nude and/or portrayed engaging in sexual activity), or if they included more than two images from the same account (to limit the potential impact of spam accounts). Any excluded post was immediately replaced. We analyzed all the content of the post, including any graphic, picture or text, as well as hashtags and text that appeared immediately below the image in the user’s caption. We did not analyze comments from other users.

We coded each variable as present, absent, or unable to code (e.g., no person or food in image to code). To assess the reliability of coding, a second coder analyzed half of all images, recording any text, additional hashtags, and the number of likes for every image. Cohen’s Kappas ranged from 0.67 to 0.98 for the variables identified (see Table 2). One author coded all 360 posts, which were then analysed.

### Statistical analysis

Statistical significance was set at *p* < 0.01 to diminish the risk of Type I error resulting from multiple analyses in this exploratory study. Chi-square analyses (or Fisher’s exact tests when expected cell sizes were five or lower) were conducted to compare the content of thinspiration versus fitspiration posts, and to compare the three SNS.

## Results

### Overall sample characteristics

Images (*N* = 360) of posts from three social media sites (*Instagram n =* 120*; Tumblr n =* 120*;* and *Twitter n =* 120) were collected. The following results describe the frequency of rated variables overall (see Table 3). Approximately three-quarters (77.2%) of the posts featured an inspirational quote or message, and 93.1% contained English text only. Of all the posts collected, 65.3% featured women, 24.0% of which displayed a sexually suggestive pose, 29.4% were shown in suggestive clothing, and 33.8% were shown partially clad. Of all posts in which a person was shown (*n* = 273), 45.0% showed the entire body, 5.5% featured before-and-after photos, 16.6% emphasized muscular features, 8.5% included posing or flexing to appear more muscular, and 16.2% showed a person engaged in physical activity. Of all the posts collected, 33.3% manipulated pose or camera angle in order to look thinner, 30.7% emphasized bony features, and 31.3% included praise for thinness. Of all posts with analyzable text in the caption (*n* = 352), 18.2% emphasized losing fat or weight, and 8.6% expressed guilt for not meeting weight or fitness goals, while 1.7% contained body positive messages. Approximately 33.8% of caption text contained messages related to reducing food intake and 21.1% included references to mental illness (eating disorders, depression or suicidality).

**Table 3:** Variable Characteristics Overall and in Each of Three Social Network Sites

Coded variable	N (%)			
	Total *N* = 360	Instagram *n* = 120	Tumblr *n* = 120	Twitter *n* = 120
Quote	82 (22.8%)	22 (18.3%)	32 (26.7%)	28 (23.3%)
Language	335 (93.1%)	109 (90.8%)	115 (95.8%)	111 (92.5%)
Image Description				
Graphic	36 (10.0%)	9 (7.5%)	20 (16.7%)	7 (5.8%)
Object	51 (14.2%)	22 (18.3%)	17 (14.2%)	12 (10.0%)
Female	235 (65.3%)	72 (60.0%)	72 (60.0%)	91 (75.8%)
Male	31 (8.6%)	15 (12.5%)	9 (7.5%)	7 (5.8%)
Male and Female	7 (1.9%)	2 (1.7%)	2 (1.7%)	3 (2.5%)
Appearance Comparison	15 (4.2%)	7 (5.8%)	6 (5.0%)	2 (1.7%)
Whole Body Visible	122 (33.9%)	41 (34.2%)	37 (30.8%)	44 (36.7%)
Head Visible	168 (46.7%)	61 (50.8%)	47 (39.2%)	60 (50.0%)
Eyes Visible	142 (39.4%)	53 (44.2%)	38 (31.7%)	51 (42.5%)
Torso Visible	249 (69.2%)	80 (66.7%)	75 (62.5%)	94 (78.3%)
Pelvis Visible	215 (59.7%)	65 (54.2%)	70 (58.3%)	80 (66.7%)
Legs Visible	201 (55.8%)	63 (52.5%)	66 (55.0%)	72 (60.0%)
Arms Visible	229 (63.6%)	72 (60.0%)	67 (55.8%)	90 (75.0%)
Body parts shown				
Partial (vs. whole) body shown	169 (62.4%)	52 (43.3%)	52 (43.3%)	65 (54.2%)
Suggestive Pose	59 (16.4%)	16 (13.3%)	22 (18.3%)	21 (17.5%)
Revealing Clothing				
Demure	90 (25.0%)	37 (30.8%)	20 (16.7%)	33 (27.5%)
Suggestive	80 (22.2%)	30 (25.0%)	26 (21.7%)	24 (20.0%)
Partially clad	92 (25.6%)	19 (15.8%)	34 (28.3%)	39 (32.5%)
Nude	10 (2.8%)	3 (2.5%)	2 (1.7%)	5 (4.2%)
Body Guilt	30 (8.3%)	16 (13.3%)	8 (6.7%)	6 (5.0%)
Weight Loss	63 (17.5%)	20 (16.7%)	23 (19.2%)	20 (16.7%)
Body Positive	6 (1.7%)	0 (0.0%)	6 (5.0%)	0 (0.0%)
Muscular Pose	23 (6.4%)	9 (7.5%)	9 (7.5%)	5 (4.2%)
Physical Activity	44 (12.2%)	12 (10.0%)	11 (9.2%)	21 (17.5%)
Muscle Emphasis	45 (12.5%)	14 (11.7%)	15 (12.5%)	16 (13.3%)
Thin Pose	91 (25.3%)	27 (22.5%)	33 (27.5%)	31 (25.8%)
Thin Praise	110 (30.6%)	30 (25.0%)	41 (34.2%)	39 (32.5%)
Bone Emphasis	84 (23.3%)	19 (15.8%)	26 (21.7%)	39 (32.5%)
Reducing Food	82 (22.8%)	40 (33.3%)	28 (23.3%)	14 (11.7%)
Mental Illness	76 (21.1%)	28 (23.3%)	35 (29.2%)	13 (10.8%)

### Fitspiration versus thinspiration posts

As #fitspiration and #fitspo content did not significantly differ on any variable assessed (*p* > 0.01), posts from these two hashtags were combined into a single group for all posts promoting fitness. See Table 4 for a summary of results. #Thinspiration, #thinspo, #thinstagram, and #thinspooo content did not significantly differ on any variable assessed with the exception of Bone Emphasis. Bone Emphasis was more highly emphasized on #thinspiration and #thinspo compared to the #thinstagram and #thinspooo hashtags on Instagram, whereas #thinspooo featured the least bone emphasis. Therefore, posts from the four thin-promoting hashtags (#thinspiration, #thinspo, #thinstagram and #thinspoooo) were analyzed together (with the exception of Bone Emphasis) as a single group.

**Table 4:** Comparison of thin vs. fit images across all three social media sites

			Images	
Coded variable	χ^2^	*p*	Thin n (%)	Fit n (%)
Quote	25.27	<.001*	21 (25.6%)	61 (74.4%)
Language	.39	.534		
All English			169 (50.4%)	166 (49.6%)
Partly English			11 (44.0%)	14 (56.0%)
Image Description	59.09	<.001*		
Graphic			12 (33.3%)	24 (66.7%)
Object			13 (25.5%)	38 (74.5%)
Female			151 (64.3%)	84 (35.7%)
Male			3 (9.7%)	28 (90.3%)
Female & Male			1 (14.3%)	6 (85.7%)
Appearance Comparison	1.87	.172	6 (40.0%)	9 (60.0%)
Whole Body Visible	25.11	<.001*	49 (40.2%)	73 (59.8%)
Head Visible	48.17	<.001*	68 (40.5%)	100 (59.0%)
Eyes Visible	49.65	<.001*	52 (36.6%)	90 (63.4%)
Torso Visible	1.26	.262	139 (55.8%)	110 (44.2%)
Pelvis Visible	.13	.721	121 (56.3%)	94 (43.7%)
Legs Visible	.39	.534	112 (55.7%)	89 (44.3%)
Arms Visible	9.58	.002*	121 (52.8%)	108 (47.2%)
Body Parts Shown				
Partial vs. Whole Body Shown	30.91	< 0.001*	69.8% partial	30.2% partial
			35.3% whole	64.7% whole
			29.2% no body	70.8% no body
			73.3% 1 part	
Number of Body Parts Shown	59.81	< 0.001*	78.3% 2 parts	26.7% 1 part
			84.6% 3 parts	21.7% 2 parts
			70.8% 4 parts	15.4% 3 parts
			48.5% 5 parts	29.2% 4 parts
				51.5% 5 parts
Suggestive Pose	.16	.688	38 (64.4%)	21 (35.6%)
Revealing Clothing	9.51	.023		
Demure			41 (45.6%)	49 (54.4%)
Suggestive			46 (57.5%)	34 (42.5%)
Partially Clad			60 (65.2%)	32 (34.8%)
Nude			8 (80.0%)	2 (20.0%)
Body Guilt	21.17	<.001*	27 (90.0%)	3 (10%)
Weight Loss	10.10	.001*	20 (31.7%)	43 (68.3%)
Body Positive	a	.030	0	6 (100%)
Muscular Pose	33.08	<.001*	0	23 (100%)
Physical Activity	53.54	<.001*	3 (6.8%)	41 (93.2%)
Muscle Emphasis	45.97	<.001*	5 (11.1%)	40 (88.9%)
Thin Pose	33.5	<.001*	74 (81.3%)	17 (18.7%)
Thin Praise	121.86	<.001*	103 (93.6%)	7 (6.4%)
Bone Emphasis	93.00	<.001*	84 (100%)	0
Reducing Food	2.46	.117	36 (43.9%)	46 (56.1%)
Mental Illness	97.56	<.001*	76 (100%)	0

* *p* < .01

^a^Fisher’s exact test was used

Coded variables Appearance Comparison (*p* = 0.172), Suggestive Pose (*p* = 0.688) and Revealing Clothing (*p* = 0.023) did not significantly differ between fitspiration and thinspiration posts. Frequency of messages promoting reduction of food intake (*p* = 0.117) also did not significantly differ between fitspiration and thinspiration posts. Body Positivity (*p* = 0.03), which was infrequently coded, did not significantly differ between thinspiration and fitspiration posts.

Thinspiration posts included more women in the image description than fitspiration, whereas fitspiration included more men, objects, and graphics than thinspiration (*p* < 0.001). The variables Body Guilt, Muscular Pose, Physical Activity and Muscle Emphasis were coded more frequently in fitspiration posts, whereas Thin Pose, Thin Praise, Bone Emphasis, and Mental Illness were coded significantly more frequently in thinspiration posts (all *p* < 0.001). The variable Muscular Pose occurred only in fitspiration images, whereas Bone Emphasis and Mental Illness occurred only in thinspiration images.

### Objectification

Fitspiration posts included more images of the entire body than thinspiration. In images that included a person, the head was visible and unobscured significantly more frequently among fitspiration posts than thinspiration posts (all *p* < 0.001). These results suggest that thinspiration posts more frequently focused on specific parts of the body, i.e., they were more objectifying.

### Fitspiration posts on Instagram vs. Tumblr vs. Twitter

There were no significant differences in fitspiration posts among Instagram, Tumblr, and Twitter on all coded variables except Body Positivity. Specifically, fitspiration posts exhibiting Body Positivity (*n* = 6) were found only on Tumblr.

### Thinspiration posts on Instagram vs. Tumblr vs. Twitter

There were no significant differences in thinspiration posts among Instagram, Tumblr, and Twitter on coded variables, except Bone Emphasis and Mental Illness (*p <* 0.001). Post hoc analyses revealed that Bone Emphasis was coded more frequently on Twitter than Instagram (*p* = 0.010). Mental Illness was coded more frequently on Tumblr than Twitter (*p* = 0.001), and on Instagram more than Twitter (*p* = 0.007), but we identified no differences between Tumblr and Instagram on any posts.

## Discussion

The present results suggest that fitspiration and thinspiration share a focus on appearance, sexually suggestive images, and restrictive eating. Although relative to fitspiration, thinspiration posts promoted thinness to a greater degree, explicitly encouraged weight loss more frequently, included more objectifying content, and made more references to eating disorders, fitspiration tended to include more messages of guilt about body shape and weight than thinspiration. When comparing fitspiration and thinspiration content between SNS, Instagram and Tumblr displayed pathological content related to eating disorders more often than Twitter.

Our study showed that fitspiration and thinspiration posts both emphasized appearance-related ideals, sexual suggestiveness (i.e., suggestive poses and revealing clothing), and restrictive eating. Similarly, Boepple & Thompson’s [11] study showed that both thinspiration and fitspiration websites showcased weight stigmatization, objectification, guilt-inducing messages about weight/body and dieting/eating restraint. Although Simpson et al. [17] looked only at fitspiration posts and included no comparison group, their study also found that most fitspiration pins on Pinterest promoted appearance-related as opposed to health-related behaviours to achieve body image standards. Another study by Boepple and colleagues [10] also showed through content analysis that fitspiration websites focused on physical appearance, eating concerns, and excessive exercise. In the current study, fitspiration displayed more messages of guilt about body shape and weight than thinspiration. This focus on appearance in fitspiration content is particularly concerning because exercising for appearance-related reasons previously has been linked to disordered eating [30], depressive symptoms [31], and negative body image [32, 33]. These problematic similarities between thinspiration and fitspiration may reflect larger changes in social conceptualizations of health and fitness, which may include mainstreaming harmful thinspiration sensibilities such as extreme food restriction and self-discipline [9]. Future qualitative research should explore people’s perceptions of differences and similarities between these two concepts, especially among SNS users.

Our findings also showed that variables related to muscularity were featured in fitspiration posts more often than in thinspiration posts. Consistent with previous research, fitspiration images in this study more frequently involved posing that emphasized muscularity, as opposed to posting pictures of people actually engaged in physical activity [16, 17, 19]. Fitspiration posts also featured images of men more frequently than thinspiration posts. Perhaps this finding is not surprising, in that male body ideals typically emphasize a muscular body shape whereas body ideals for women place greater emphasis on thinness [34]. A meta-analysis has shown that exposure to muscular ideals worsens men’s body image [35]; the relationship between muscularity and body image, however, may be more complex for women [21]. In light of previous research findings, it is plausible that fitspiration posts focusing on muscular ideals for men could contribute to body dissatisfaction, although more research is needed to understand its effects in men versus women. The increasing popularity of fitspiration and the fit-ideal messages like “Strong is the new skinny” suggest that the thin-ideal for women is shifting to incorporate a fit-ideal similar to men’s association of muscularity with physical attractiveness. Given the rapid pace of change in social media trends, it seems important to track and monitor these ideal body shape trends for both men and women further, in the event that the fit-ideal becomes a pathological concern like the thin-ideal has been for women in recent decades.

The current study showed that thinspiration posts promoted thinness, explicit encouragement of weight loss, objectifying content, and made references to eating disorders more often than fitspiration, similar to previous [11] findings. Notably, emphases on bony features and mental illness were present only in thinspiration posts. Fitspiration posts tended to be less objectifying (i.e., they depicted more whole-body images) than thinspiration posts, which displayed more objectifying content (i.e., they depicted more specific body parts).

We detected no systematic differences in fitspiration content among the three SNS, other than body positivity being found only on Tumblr. On the other hand, thinspiration content differed among the three SNS. References to mental illness (i.e., related to eating disorders) were more frequent on Tumblr and Instagram than on Twitter, and bone emphasis in thinspiration posts was coded more frequently on Twitter than on Instagram. Note that both Tumblr and Instagram included warning displays with information on eating disorder resources when the hashtags #thinspiration and #thinspoooo were searched on either site. Perhaps there were fewer references to mental illness on Twitter because of its character limit on text posts, whereas Instagram and Tumblr do not have such limits. These differences may also reflect the popularity of certain SNS among thinspiration or pro-eating disorder communities; Twitter places the least emphasis on photo content of the three SNS and may therefore be less popular for these users.

The following study limitations should be considered when interpreting our results. As content on these sites is quite fluid, it is unclear how stable our study findings would be in future research, although it may be important to assess. We did not capture images that promoted thinspiration and/or fitspiration if they did not use any of the six hashtags we searched for. Furthermore, we searched for only two fitspiration hashtags (#fitspiration and #fitspo) and two thinspiration hashtags (#thinspiration and #thinspo on Tumblr and Twitter, #thinspoooo and #thinstagram on Instagram) on each SNS. We limited our hashtag searches to the most commonly used hashtag variations of #thinspiration and #fitspiration and did not analyze other hashtag variations, such as #fitspirational, #fitspirations and #fitspirationfriday. Our search was additionally restricted to posts drawn from just four days in total. Given these limitations, our analysis provides only a snapshot of a broad and continuously updated body of content across social media. We chose Instagram, Tumblr and Twitter primarily for their emphasis on photo sharing; previous research has examined other SNS, such as Pinterest and WeHeartIt [7, 24, 26]. The content of dedicated pro-eating disorder groups has also been examined on Facebook [8], which can also be used to share images. However, the Facebook interface does not emphasize public photo sharing in the same way as the SNS we studied. Although Facebook is the most widely used SNS [36], other sites such as Instagram are approaching the same level of use among teens, and were recently ranked as more preferred by teens [37]. Instagram displayed a warning page when #thinspoooo was searched, and displayed the most popular images, rather than the most recent. For all other hashtag searches, however, we collected the most recent posts. Thus, the Instagram posts we captured may have systematically differed from the other two SNS. Future research would benefit from the use of external search engines such as Google Advanced Search or Social Searcher to avoid these limitations.

Given the evolving nature of social media usage, future research is needed to continue monitoring its content and investigate experimentally its potential effects on body image, body dissatisfaction, eating and exercising patterns, and other symptoms of mental illness. Fitspiration is a relatively new phenomenon, and future research should monitor changes in fitspiration content, especially given the emphasis on fitness in mainstream culture. If thinspiration and fitspiration are deemed harmful, it would be important to create ways to discourage such posts, similar to the thinspiration warning messages found on Instagram and Tumblr. Moreover, we do not know the long-term effects of creating, viewing, and using SNS content displayed in fitspiration messages. It would be useful to conduct experimental research on the effects of creation of, and exposure to, fitspiration posts on the mental health of social media creators and users, in all genders, in the short and long term.

## Conclusions

The present results suggest that fitspiration and thinspiration display many similarities, especially a focus on appearance, sexual suggestiveness, and restrictive eating. Thinspiration and fitspiration found on different SNS should continue to be monitored. Future experimental studies should examine the effects of creation, viewing, and usage of thinspiration and fitspiration content on the mental health of their creators, viewers, and users.
